# Caries Experience and Treatment Needs in Urban and Rural Environments in School-Age Children from Three Provinces of Ecuador: A Cross-Sectional Study

**DOI:** 10.3390/dj10100185

**Published:** 2022-10-01

**Authors:** Eleonor María Vélez-León, Alberto Albaladejo-Martínez, Katherine Cuenca-León, Liliana Encalada-Verdugo, Ana Armas-Vega, María Melo

**Affiliations:** 1 Department of Surgery, Faculty of Medicine, University of Salamanca, 37007 Salamanca, Spain; 2 School of Dentistry, Catholic University of Cuenca, Cuenca 010107, Ecuador; 3 School of Dentistry, Hemisferios University, Quito 170527, Ecuador; 4 Department of Stomatology, Faculty of Medicine and Dentistry, University of Valencia, 46010 Valencia, Spain

**Keywords:** epidemiology, caries prevalence, caries experience

## Abstract

In Ecuador, national epidemiological surveys have not been updated; however, some regional studies in the northern areas of the country still report a high prevalence of dental caries. The aim of this study was to determine the experience, severity, and need for treatment of dental caries in school children aged 6 to 12 years in urban and rural settings in three provinces of southern Ecuador. This cross-sectional, relational study examined 1938 schoolchildren in the provinces of Azuay, Cañar, and Morona Santiago. The survey instruments were based mainly on the WHO manual Methods of Oral Health Surveys (dmft) for primary and permanent dentition (DMFT), as well as the prevalence, severity, and Significant Caries Index (SCI). The parametric Student’s t-test was used to compare two groups, and the Spearman’s Rho and Tau-c Kendall correlation coefficients were used to associate the categorical variables. Results: The prevalence of caries in the primary dentition was 78% and 89.2% in the permanent dentition. The dmft (M = 4.12, SD = 2.86) and DMFT (M = 3.62: SD = 3.07) placed the general group in a moderate caries index. The need for treatment was 90.68% in the primary dentition, while it was 87.99% in the permanent dentition. Caries severity in both dentitions was high (M = 7.74; SD = 3.42). Conclusions. Alarming indicators of caries experience and the need for treatment were observed in the population studied.

## 1. Introduction

Dental caries continues to be one of the most prevalent oral diseases worldwide. The etiology is multifactorial, with biological, genetic, behavioral, and social modifying factors [1,2,3]. Diet and feeding practices are essential in the development of caries [4,5]. Globally, there are significant variations in caries experience between and within countries, especially in school and pre-school children, reflecting the influence of different risk factors on the presence of this disease [6,7]. In children, the state of oral health also depends on parental factors, as, until the first 5 years of age, children acquire dietary and oral hygiene practices from their parents and/or caregivers [8,9,10,11]. In this context, it is important that the school environment reinforces the practices acquired at home and that oral health policies are implemented as recommended by the World Health Organization (WHO) [12,13,14].

In epidemiological studies, an important factor to study is the “environment”; that is, whether the place of residence belongs to the urban or rural area, as this could generate inequities in education and health [15,16]. In addition, rural areas, due to their demographic characteristics, are associated with certain conditions where income is lower and access to health services is limited [15,17,18,19]. In certain studies, it has even been observed that the rural environment, when differentiated from urban communities by a higher poverty rate, could add stress to parents and affect their feeding and hygiene practices in raising their children [17]. These conditions could increase the risk of developing oral diseases in children and adolescents.

In Ecuador, public health measures for the prevention of dental caries have been based on a single epidemiological study developed in 1996, and on an oral health report in 2009, which reported a high incidence of oral problems, such as caries, gingivitis, and dental fluorosis, in both children and adults [18,19]. In 1996, the prevalence of dental caries in school children between 6 and 15 years of age registered a value of 88.2%, the need for dental treatment in 14.8%, the frequency of dental surgery procedures reached 85.2%, exodontia was 16.9%, and endodontics was 10.5% [18]. By 2009, the prevalence of dental caries in school children examined in the same age range decreased to 75.6% [19].

More recent studies from different regions of the country with non-representative population samples have reported a prevalence of 70% of caries in children between 8 and 10 years of age [20,21,22]. These high rates are related to the level of social, economic, and educational inequality in the country [23]. As can be seen in the studies mentioned above, the prevalence of dental caries is higher than 50% in the entire population, and even more in school children who are a vulnerable group, due to their diet and lack of interest in tooth brushing, as well as a lack of knowledge about the importance of dental care [3,24]. The provinces of Azuay, Cañar, and Morona Santiago are located in the southern region of Ecuador. This area is characterized by a Multidimensional Poverty Index (MPI) of 47.4, which exceeds the countrywide MPI of 16.9; these values are associated with deficiencies in the indicators of water, health, education, food, and housing [25], so it is important to update the information on the situation of dental caries experience and the need for treatment according to age, sex, and environment in this population.

## 2. Materials and Methods

### 2.1. Study Design

An observational, relational cross-sectional epidemiological study was proposed, respecting the international standards of the Declaration of Helsinki made under the approval of the Board of Directors of the Academic Unit of Health and Welfare with the code No048 C.D2019 (14 February 2019). The authorities and representatives of the children were informed about the study and provided informed consent.

### 2.2. Variables

**Figure 1:**
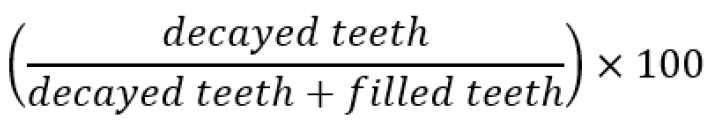
Formula for treatment requirement.

The diagnosis of carious lesions was made using the ICDAS II criteria, at cut-off point of scores 3–6, as it was found that the use of this combined format in studies carried out in preschool children, school children, and adolescents minimized the difference between the WHO standard and the ICDAS-II criteria [26]. A tooth with a filling and carious lesion or temporary filling was also considered a carious tooth (Dt). The tooth was recorded as missing (Mt) when it was extracted due to complications of the carious process (verified by interview). When any doubt arose as to the reason for extraction, the missing tooth was not included in the index. The tooth was assumed to be filled (Ft) when at least one permanent restoration was placed for the treatment of carious lesions. The methodology used was found in other research [26,27,28,29]. Based on these criteria, the following indicators were established:

- Caries experience, also known as dental morbidity, refers to the rate of decayed, filled, and missing teeth due to caries and dmft/DMFT indices [28].
- Caries prevalence, which refers to the percentage of affected individuals, was coded for analysis as “no caries” if DMFT = 0 and “with caries” if DMFT > 0.
- Caries severity, which refers to the classification of caries according to its severity, was coded as “no caries” if DMFT + dmft = 0, “low severity” if DMFT + dmft ≤ 3, “medium severity” if DMFT + dmft = between 4 and 6, and “high severity” if DMFT + dmft ≥ 7 [30].
- Significant caries index (SIC), which refers to the average rate of DMFT in the third of the population most affected by caries [31].
- Treatment needs [32], which was obtained with the following formula (Figure 1):

### 2.3. Population and Sample

According to the report of the National Institute of Statistics and Census (2010 report) [33], the estimated population of children between 6 to 12 years of age was 183,081, so the sample size was calculated for convenience using EPIDAT 4.0, resulting in 1938 participants.

In order for the schoolchildren to be part of the research, it was necessary to meet the following inclusion criteria: age from 6 to 12 years, belonging to the residence environments selected for the study, having consent signed by the legal representative, not having systemic impediment or the use of fixed devices that prevented the examination, and not having a systemic impediment or the use of fixed devices that prevented the examination.

### 2.4. Calibration

The calibration analysis was performed at the tooth level, using the dmft/DMFT index configuration, for primary and permanent dentition, respectively, with a diagnostic cut-off point at the level of code 3 level of the ICDAS II criteria [34]. This process was conducted by certified professionals in the field and consisted of three theoretical sessions, with practical exercises using clinical images and extracted teeth with carious lesions, followed by two group clinical sessions with 10 school children of each age group from a local institution. In the clinical part of the calibration process, each examiner reviewed the two groups of children accompanied by a dental student who would assist him/her in recording the information on the forms. The concordance between examiners according to Cohen’s kappa was 0.83 for the primary dentition and 0.86 for the permanent dentition.

### 2.5. Examination

Prior to the examination, the children brushed their teeth under the supervision of dental students. The examinations were carried out in spaces provided by the school staff under conditions standardized by the WHO [35]. The children were examined in chairs with straight backs, and each examiner had artificial light, cotton rolls, and gauze for humidity control. All surfaces were examined and caries were diagnosed visually according to the ICDASII criteria [36]. The calibrated examiners examined while the assistants completed the data collection form.

### 2.6. Statistical Analysis

The data obtained were recorded in previously prepared forms, according to the guidelines published by the WHO [37]. The results were initially expressed through measures of central tendency and dispersion, and later, the classifications were executed using percentage frequencies. The behavior of the data was normal using the Kolmogorov–Smirnov test (*p* > 0.05), so the parametric test was used for the comparison of two Student’s t-tests (T). To associate the categorical variables in 5 × 2 rectangular matrices (DMFT and dmft with five categories and sex and age with two categories), Spearman’s Rho and Kendall Tau-c correlation coefficients were used. The data analysis was performed in the statistical program SPSS V28 IBM®SPSS v.27 (New York, NY, USA) and JASP®0.16.2 (Amsterdam, The Netherlands) statistical programs were used and 0.05 was considered statistically significant (*p* < 0.05).

## 3. Results

The final sample consisted of 1938 students, 997 males (51.4%) and 941 females (48.6%), with 48.0% from an urban setting and 52.0% from a rural setting. The distribution of participants by province can be seen in Table 1.

At the general level of the sectors studied, the presence of caries was recorded in 93.3% of the school children. In the primary dentition, this was 78%, and in the permanent dentition it was 89.2%. The dmft (M = 4.12, SD = 2.86) and DMFT (M = 3.62; SD = 3.07) placed the general group in a moderate caries index. The need for treatment was 90.68% in the primary dentition, while it was 87.99% in the permanent dentition. Caries severity in both dentitions was high (M = 7.74; SD = 3.42), and there was heterogeneity of behavior; 0.7% were caries-free, 10.3% had low severity, 26.9% had medium severity, and 62.2% had high severity.

The SIC index ranged from 4 to 14 (M = 7.21), with a mean data dispersion (SD = 2.3) well above 3, which implies severity in the presence of caries.

Regarding caries indicators according to sex, between 19 and 22% of children presented at least some restoration in the primary dentition, and the rate of restoration per child being very low (0.6–0.7). Between 41 and 45% of school children were already missing at least one tooth due to caries. Females had more experience of caries in the primary dentition and the dmft index was similar for both sexes.

In the permanent dentition, there were no statistically significant differences in the individual indicators, but the SIC index was higher in males (Table 2).

The results of caries severity in males revealed that 0.8% were caries-free, 10.8% had low severity, 27.6% had moderate severity, and 60.8% had high severity, compared with 0.6% of females who were caries-free, 9.7% with low severity, 26.2% with medium severity, and 63.5% with high severity with no significant differences (X^2^ = 2.924; *p* = 0.404).

In reference to the residential environment of the participants, the only difference found was in the indicator of missing pieces of the primary dentition, which was higher in rural areas (*p* < 0.05), with high rates of dmft/DMFT in both environments. Environment was not recorded as a factor related to caries severity (X^2^ = 1.667, *p* = 0.646). Here, 0.6% and 0.7% of participating schoolchildren were caries-free in urban and rural settings, respectively; 11.3% and 9.1% had low severity, respectively; 26.1% and 27% had medium severity, respectively; and 62.0% and 62.4% had high severity, respectively (Table 3).

It was identified that the number of healthy, carious, and filled primary teeth decreased as the age increased. The dmft index was also higher in 6-year-old children. The need for treatment did not show a correlation; the age group with the greatest need for treatment were children 12 years of age. The average number of healthy, carious, and filled teeth in the permanent teeth increased gradually as the age increased (*p* < 0.05). The need for treatment of the primary teeth ranged from 82.53% to 95.87% (Table 4).

## 4. Discussion

The findings reveal a high prevalence and severity of caries in the evaluated population, especially in rural areas, coinciding with previous studies conducted in Ecuador and in other populations, such as Chile, where similar data were reported in 12-year-old children, which showed an increase in the presence of carious lesions directly proportional to the age of the participants [15,18,20,38], observing how the area of residence influences health indicators, due to access to health services [30] and the presence of specialized medical personnel [15]. However, it is evident that the multifactorial nature of dental caries disease cannot be assessed only by this variable, but also by the nutritional status [39,40] and socioeconomic inequalities [41,42], which, when combined with the area of residence, can trigger disease and thus a decrease in the quality of life.

Epidemiological studies carried out in populations in Europe (North and West) and the United States show a decrease in the prevalence of carious lesions [43,44,45] in 12-year-old participants, with CPOD indexes were found of 1 in France and Germany, 1.3 in Spain, and 1.5 in the United Kingdom and Portugal [46,47,48], which indicate the evident effectiveness of the actions that the health entities have exercised in these populations, with strategies established from the first years of life [49], with emphasis on the education directed to parents, caregivers, and children, as well as on the preventive processes and plans [27,49]. Different studies have shown that there are predominant disease patterns depending on the sector [6,32,44], which are related to greater knowledge of health issues and greater access to health services among mothers living in urban areas, but also greater access of their children to processed foods [6,23,50].

The geographical limitation of the population evaluated, with specific socioeconomic and cultural characteristics, is limited to a certain area and does not manage to represent the total population of the country; however, the results found allow for extrapolating and analyzing the health status of the Ecuadorian population. Another element that may constitute a limitation is the cross-sectional assessment that was performed, as the analysis of the oral health status was carried out in a certain period of time and the actions carried out in the population could not be analyzed. The fact that different studies have been carried out in Ecuador analyzing the state of oral health, and that each one of them presents different protocols [51], makes it difficult to analyze and compare the results, which invites us to reflect on the need for structured national studies with standardized methodologies that allow for evaluating their effects.

As clinicians, we face the daily challenge of controlling oral problems, where caries are the most frequent; however, the multicultural nature of the Ecuadorian population becomes a challenge that should be considered in the control strategies to be implemented, where education for all stakeholders, the population, and health professionals should become one of the immediate action measures to be implemented at all levels of health care.

## 5. Conclusions

The school children in the urban and rural areas of the population studied here presented high values of caries experience, as well as a significant need for treatment.

Although there may be several social determinants that could explain these results, we recommend further studies to confirm these findings, which could serve as a starting point for future research in the field of oral epidemiology, an area that is not very updated in Ecuador.

## Figures and Tables

**Table 1:** Distribution of the population according to age, sex, and province.

Characteristic		Azuay (*n* = 667)				Cañar (*n* = 754)				Morona Santiago (*n* = 517)			
		Urban (*n* = 380)		Rural (*n* = 287)		Urban (*n* = 276)		Rural (*n* = 478)		Urban (*n* = 275)		Rural (*n* = 242)	
		*n*	%	N	%	*n*	%	*n*	%	*n*	%	N	%
Sex	Men	194	51.1	148	51.6	155	56.2	252	52.7	147	53.5	101	41.7
	Women	186	48.9	139	48.4	121	43.8	226	47.3	128	46.5	141	58.3
Age (years)	Six	70	18.4	65	22.6	53	19.2	89	18.6	40	14.5	54	22.3
	Seven	52	13.7	36	12.5	48	17.4	58	12.1	30	10.9	33	13.6
	Eight	53	13.9	38	13.2	33	12.0	42	8.8	45	16.4	51	21.1
	Nine	52	13.7	38	13.2	30	10.9	62	13.0	40	14.5	37	15.3
	Ten	51	13.4	38	13.2	34	12.3	50	10.5	40	14.5	36	14.9
	Eleven	51	13.4	38	13.2	35	12.7	67	14.0	40	14.5	15	6.2
	Twelve	51	13.4	34	11.8	43	15.6	110	23.0	40	14.5	16	6.6

**Table 2:** Caries experience and treatment needs in urban and rural environments according sex.

		Primary Dentition						Permanent Dentition					
		N	% (IC)	X (*p*)	Mean	SD	t (*p*)	N	% (IC)	X (*p*)	Mean	SD	t (*p*)
Carious	M	816	81.8 (79.4–84.1)	0.956 (0.328)	3.1	2.6	−0.932 (0.351)	827	82.9 (80.5–85.2)	0.837 (0.360)	3.3	3.1	1.645 (0.100)
	W	786	83.5 (81.1–85.8)		3.3	2.7		795	84.5 (82.1–86.7)		3.1	2.7	
Filled	M	197	19.8 (17.4–22.3)	1.756 (0.185)	0.3	0.6	−2.117 (0.034 *)	276	27.7 (25.0–30.5)	0.056 (0.814)	0.4	0.8	0.032 (0.974)
	W	209	22.2 (19.6–24.9)		0.4	0.7		256	27.2 (24.4–30.1)		0.4	0.8	
Missing	M	413	41,4 (38.4–44.5)	3.959 (0.047 *)	0.6	0.9	−2.08 (0.038 *)	18	1.8 (1.1–2.8)	0.301 (0.583)	0	0.2	0.943 (0.346)
	W	432	45.9 (42.7–49.1)		0.7	0.9		14	1.5 (0.92.4)		0	0.1	
Need for treatment (%)	M	984	98.7 (97.8–99.3)	1.140 (0.286)	91.5	18.8	1.605 (0.109)	967	97.0 (95.8–97.9)	0.240 (0.624)	88.2	22.6	0.443 (0.658)
	W	923	98.1 (97.1–98.8)		89.9	21.1		909	96.6 (95.3–97.6)		87.7	23.7	
dmft/DMFT > 1	M	874	87.7 (85.5–89.6)	4.119 (0.042 *)	4.0	2.9	−1.993 (0.046 *)	857	86.0 (83.7–88.0)	1.764 (0.184)	3.7	3.3	1.604 (0.109)
	W	852	90.5 (88.5–92.3)		4.3	2.9		828	88.0 (85.8–90.0)		3.5	2.9	
SIC	M	M = 7.48 (DE = 2.4)						T = 3.249 *p* = 0.001					
	W	M = 6.89 (DE = 2.2)											
Caries severity (dft + DMFT > 1)	M	M = 7.73 (DE = 3.51)						t = −0.23 *p* = 0.818					
	W	M = 7.76 (DE = 3.34)											

Note: * *p* < 0.05 (Significant difference). SD = standard deviation. U: urban. A: rural. dmft/DMFT: rate of caries. Missing and filled. M = male. W = women.

**Table 3:** Caries experience and treatment needs according to urban/rural setting.

		Primary Dentition						Permanent Dentition					
		N	% (IC)	X	Mean	SD	T	N	% (IC)	X	Mean	SD	t
				(*p*)			(*p*)			(*p*)			(*p*)
Healthy	U	783	84.1 (81.7–86.3)	0.576	6.4	4.7	−0.513 (0.607)	851	91.4 (89.5–93.1)	1.024 (0.312)	10.2	6.3	0.151 (0.880)
	R	834	82.8 (880.4–85.1)	(0.488)	6.5	4.8		933	92.7 (90.9–94.1)		10.2	6.8	
Carious	U	773	83.0 (80.5–85.3)	0.168	3.2	2.7	0.539 (0.590)	767	82.4 (79.8–84.7)	2.253 (0.133)	3.2	3	0.150 (0.881)
	R	829	823 (79.9–84.6)	(0.682)	3.2	2.6		855	84.9 (82.6–87.0)		3.2	2.8	
Filled	U	193	20.7 (18.2–23.4)	0.052	0.3	0.6	−0.779 (0.436)	268	28.8 (25.9–31.8)	1.604 (0.205)	0.4	0.8	1.161 (0.246)
	R	213	21.2 (18.7–23.8)	(0.820)	0.3	0.7		264	26.2 (23.6–29.0)		0.4	0.7	
Missing	U	380	40.7 (37.7–44.0)	5.653	0.6	0.9	−2.406 (0.016 *)	14	1.5 (0.9–2.4)	0.240 (0.624)	0	0.2	−0.256 (0.798)
	R	465	46.2 (43.1–49.3)	(0.017 *)	0.7	0.9		18	1.8 (1.1–2.7)		0	0.1	
Need for treatment (%)	U	918	98. 6 (97.7–99.2)	0.47	91.1	19.1	0.827 (0.408)	898	96.5 (95.1–97.5)	0.690 (0.406)	87.2	23.9	−1.321 (0.187)
	R	989	98.2 (97.3–98.9)	(0.493)	90.3	20.7		978	97.1 (95.9–98.0)		88.7	22.5	
dmft/DMFT > 1	U	819	88.0 (85.8–89.9)	2.189	4.1	2.9	−0.434 (0.664)	801	86.0 (83.7–88.1)	1.304 (0.254)	3.7	3.2	0.417 (0.676)
	R	907	90.1 (88.1–91.8)	(0.139)	4.2	2.8		884	87.8 (85.7–89.7)		3.59	2.98	
SIC	U	M = 7.35 (DE = 2.42)						t= 1.46					
	R	M = 7.09 (DE = 2.25)						*p* = 0145					
Caries severity	U	M = 7.75 (DE = 3.55)						t = −0.011					
(dmft + DMFT > 1)	Rural	M = 7.74 (DE = 3.30)						*p* = 0.992					

Note: * *p* < 0.05 (Significant difference). SD = standard deviation. U: urban. A: rural. dmft/DMFT: rate of caries. Missing and filled. M = male. W = women.

**Table 4:** dmft/DMFT according to age (years).

Indicators			Age							Rho Spearman (*p*)
			Six	Seven	Eight	Nine	Ten	Eleven	Twelve	
Primary	Healthy	mean	12.5	10	7.5	6.1	4.8	2.1	0	−0.899 (<0.01 *)
		SD	3.1	2.8	2.4	2.1	1.6	1.4	0.1	
	Carious	mean	5.1	4.8	3.8	3.3	2.4	2.3	0.2	−0.609 (<0.01 *)
		SD	3	2.7	2.3	1.9	1.6	1.4	0.5	
	Filled	mean	0.4	0.3	0.4	0.3	0.1	0.4	0	−0.128 (<0.01 *)
		SD	0.8	0.6	0.8	0.6	0.4	0.6	0.1	
	Missing	mean	0.5	0.6	0.4	0.9	1	0.9	0.3	−0.084 (<0.01 *)
		SD	1	0.9	0.7	0.9	1	1	0.5	
	Need for treatment	mean	92	92	89.3	91.1	93.3	84	98.3	−0.011 (0.668)
		SD	18.2	16.6	21.2	18.9	19.6	24.9	13	
	dmft	mean	6	5.7	4.5	4.5	3.5	3.6	0.5	−0.569 (<0.01) *
		SD	3.1	2.8	2.4	2.2	1.9	1.7	0.7	
Permanent	Healthy	mean	0.9	6.2	8.3	10.5	12.8	16.4	19.3	0.944 (<0.01) *
		SD	1	1.4	1.8	2.1	2.4	2.8	3.6	
	Carious	mean	1	1.5	2.3	3.1	3.8	5.2	6.3	0.641 (<0.01) *
		SD	1	1.2	1.7	1.9	2.1	3	3.5	
	Filled	mean	0	0.2	0.3	0.3	0.4	0.6	1	0.385 (<0.01)
		SD	0.2	0.5	0.6	0.6	0.7	0.8	1.2	
	Missing	mean	0	0	0	0	0	0	0	0.027 (0.232)
		SD	0.1	0.1	0	0.2	0.2	0.2	0.2	
	Need for treatment (%)	mean	95.9	84.8	88.1	91.5	87.7	86.5	82.5	−0.281 (<0.01)
		SD	18.4	31.4	25.4	18.9	22.2	21	21.4	
	DMFT	mean	1.1	1.7	2.6	3.4	4.2	5.8	7.3	0.704 (<0.01) *
		SD	1	1.3	1.7	2	2.2	2.9	3.4	
Caries severity		mean	7.1	7.4	7.1	8	7.8	9.4	7.9	0.145 (<0.01)
		SD	3.4	3.3	3.2	3.4	3.3	3.4	3.4	

Note: * *p* < 0.05 (significant relationship). SD = standard deviation.

## Data Availability

https://ucacueedu-my.sharepoint.com/personal/mvelezl_ucacue_edu_ec/_layouts/15/onedrive.aspx?ga=1&id=%2Fpersonal%2Fmvelezl%5Fucacue%5Fedu%5Fec%2FDocuments%2FBase%20nueva%20Eleonor%20Velez%20%281%29%2Esav&parent=%2Fpersonal%2Fmvelezl%5Fucacue%5Fedu%5Fec%2FDocuments&p=14 (accessed on 18 December 2021).
